# Hospitalizations for acetaminophen overdose: a Canadian population-based study from 1995 to 2004

**DOI:** 10.1186/1471-2458-7-143

**Published:** 2007-07-05

**Authors:** Robert P Myers, Bing Li, Andrew Fong, Abdel Aziz M Shaheen, Hude Quan

**Affiliations:** 1 Liver Unit, Division of Gastroenterology, Department of Medicine, University of Calgary, Calgary, Alberta, Canada; 2 Department of Community Health Sciences; University of Calgary, Calgary, Alberta, Canada

## Abstract

**Background:**

Acetaminophen overdose (AO) is the most common cause of acute liver failure. We examined temporal trends and sociodemographic risk factors for AO in a large Canadian health region.

**Methods:**

1,543 patients hospitalized for AO in the Calgary Health Region (population ~1.1 million) between 1995 and 2004 were identified using administrative data.

**Results:**

The age/sex-adjusted hospitalization rate decreased by 41% from 19.6 per 100,000 population in 1995 to 12.1 per 100,000 in 2004 (*P *< 0.0005). This decline was greater in females than males (46% vs. 29%). Whereas rates fell 46% in individuals under 50 years, a 50% increase was seen in those ≥ 50 years. Hospitalization rates for intentional overdoses fell from 16.6 per 100,000 in 1995 to 8.6 per 100,000 in 2004 (2004 vs. 1995: rate ratio [RR] 0.49; *P *< 0.0005). Accidental overdoses decreased between 1995 and 2002, but increased to above baseline levels by 2004 (2004 vs. 1995: RR 1.24;*P *< 0.0005). Risk factors for AO included female sex (RR 2.19; *P *< 0.0005), Aboriginal status (RR 4.04; *P *< 0.0005), and receipt of social assistance (RR 5.15; *P *< 0.0005).

**Conclusion:**

Hospitalization rates for AO, particularly intentional ingestions, have fallen in our Canadian health region between 1995 and 2004. Young patients, especially females, Aboriginals, and recipients of social assistance, are at highest risk.

## Background

Acetaminophen is the most commonly implicated drug in cases of acute liver failure (ALF) predominantly due to its widespread availability [1-5]. Excluding combination preparations, approximately 1.5 billion tablets are sold annually in Canada[6]. Recent data from the US ALF Study Group identified acetaminophen as the etiology in approximately 50% of cases[2,3]. Acetaminophen overdose typically has a good prognosis, even if hepatic failure has developed. Less than 5% of patients who take toxic quantities of acetaminophen (approximately 150 mg/kg body weight) develop acute liver toxicity [7]. and survival without transplantation for those who develop encephalopathy (~65%) exceeds that for most other forms of ALF [2-4]. Nevertheless, nearly one-third of those developing encephalopathy will die and 8% require transplantation [2-4]. The cost of treating patients with acetaminophen overdose was estimated at over $87 million annually in the US in 1995;[8] this is likely a conservative estimate in light of current health care costs.

Due to these important public health implications, a wealth of literature has focused on the epidemiology of acetaminophen overdose [9-19]. Most reports originate in the United Kingdom (UK) where legislation in 1998 limited the size of packets of acetaminophen to 16 tablets of 500 mg[9,10]. Although results are variable, most studies suggest a benefit of this legislation. In a systematic review examining the impact of these restrictions, Morgan *et al*. reported a reduction in hospital admissions and liver transplants for acetaminophen overdose, but conflicting findings regarding the severity of poisonings, deaths, and over the counter sales following this legislation[11].

Recently, Prior *et al*. examined the epidemiology of acetaminophen overdose in Canada[6]. Between 1995 and 2002, the annual hospitalization rate for acetaminophen overdose was 27 per 100,000 population. This rate did not change significantly after the lifting of restrictions limiting sales of acetaminophen (tablets > 325 mg or quantities > 24 tablets) to pharmacies only in 1999–2000[6]. Unfortunately, this study did not describe the characteristics of patients with acetaminophen overdose, including sociodemographic factors. Studies have suggested that younger age and female gender increase the risk of acetaminophen overdose,[12,13] but Canadian data is lacking.

Therefore, we conducted a population-based study to examine risk factors for acetaminophen overdose in a large Canadian health region. We were particularly interested in the impact of Aboriginal race and low socioeconomic status on rates of acetaminophen overdose since a wealth of literature has demonstrated greater health disparities, including an increased risk of suicide, in Aboriginal [14,15] and lower income Canadians[16,17]. We also examined temporal trends in hospitalization rates for acetaminophen overdose, including both intentional and accidental ingestions. An understanding of the predisposing factors for acetaminophen overdose might permit targeting of preventive initiatives at high risk subgroups if a substantial need is uncovered by the epidemiologic analysis.

## Methods

### Study population

The study population consisted of hospitalized patients with acetaminophen overdose residing in the Calgary Health Region (CHR) between fiscal years 1995 to 2004. The CHR provides virtually all medical and surgical care to approximately 1.1 million residents of Calgary and surrounding communities in the southern part of the province of Alberta. Hospitalized acetaminophen overdose cases were identified using CHR hospital discharge abstract administrative data. This database contains 16 diagnosis and 10 procedure coding fields. Cases of acetaminophen overdose were identified via a search of the 16 diagnosis coding fields for diagnostic code 965.4 according to the *International Classification of Disease, Ninth Revision, Clinical Modification *(*ICD-9-CM*) [18] in the 1995–2001 discharge data, and T39.1 according to *ICD-10 *[19] in the 2002–2004 discharge data. Trained health records nosologists at each hospital code all diagnoses prior to submission of the data to Alberta Health and Wellness. Coding is based on the entire hospital record. Non-residents of the CHR were excluded. The first admission was assigned as the index admission for patients with repeated hospitalizations for acetaminophen overdose.

### Definitions of variables

The identified acetaminophen overdose cases were linked with the Alberta Health Care Insurance Plan (AHCIP) Registry [20]. using a unique personal health number. Since the ACHIP is a government-administered universal plan providing health care for over 99% of Albertans, this registry includes nearly all residents of Alberta[20]. Demographics and information regarding Aboriginal and socioeconomic status (SES) were extracted from this registry. Aboriginal status was ascertained using a field that identifies individuals with "Treaty status" based on treaties between their First Nations bands and the federal government, which entitle patients to comprehensive health care without insurance premiums[20,21]. As a proxy for low SES, the receipt of social assistance or an insurance premium subsidy from Alberta Health and Wellness were examined. In 2004, 2% of Albertans received social assistance and 13% received a subsidy. All individuals 65 years of age and older (herein referred to as seniors) are eligible for this subsidy regardless of income. Due to the methods of coding in the AHCIP Registry, receipt of a subsidy or social assistance could not be determined in status Aboriginals.

Comorbid depression was defined using *ICD-9-CM *and *ICD-10 *coding algorithms developed by Quan *et al*. [22]. Alcohol-related diagnoses were defined using a previously-validated *ICD-9-CM *diagnosis coding algorithm for discharge data from fiscal years 1995–2001[23,24]. We translated *ICD-9-CM *codes to corresponding *ICD-10 *codes for 2002–2004 discharge data via a search of both coding manuals. All 16 diagnosis fields were used to define these comorbidities.

The circumstance in which the overdose occurred was classified as accidental (*ICD-9-CM*, E850.4, E935.4; *ICD-10*, X40, Y45.5), intentional (*ICD-9-CM*, E950.0;*ICD-10*, X60), or other (including homicidal [*ICD-9-CM*, E962;*ICD-10*, X85] and undetermined intent) using 'external causes of injury' codes (E-codes). These codes are frequently used to define injuries according to mechanism (eg. poison, firearm, motor vehicle) and intent (eg. accidental, suicide, assault)[25]. Any overdose involving self-inflicted poisoning by another drug, medicinal or biological substance in addition to acetaminophen (*ICD-9-CM*, E950.1-E952.9;*ICD-10*, X61-X69) was considered intentional.

### Statistical methods

Descriptive statistical methods were used to describe demographic and clinical characteristics of patients with acetaminophen overdose at their first hospitalization. We also compared patients with single versus multiple hospitalizations to identify characteristics that might be particularly strong risk factors for acetaminophen overdose. Between group comparisons were made using Fisher's exact, χ^2^, and Mann-Whitney tests, as appropriate. Hospitalization rates were calculated by considering the entire end-of-fiscal year population of the CHR (as obtained from the AHCIP Registry) as at risk. Direct age/sex-adjusted rates were calculated using the 2001 Canadian population as the standard. Temporal trends in hospitalization rates and selected risk factors (age, gender) for acetaminophen overdose were evaluated using Poisson log-linear regression[26]. The impact of Aboriginal status, and receipt of social assistance or an insurance premium subsidy on rates of acetaminophen overdose were determined by comparing the prevalence of these indicators among acetaminophen overdose cases versus the remainder of the population using Fisher's exact test.

The study protocol was approved by the Conjoint Health Research Ethics Board at the University of Calgary.

## Results

### Study population

Between 1995 and 2004, 1,543 patients had 1,680 admissions for acetaminophen overdose. The majority (68%) was female and the median age at first hospitalization was 26 years (interquartile range [IQR], 2–85) (Table 1). Females were significantly younger than males at presentation (median age [IQR]: 24 [12–84] vs. 29 [2–76] years; z = -5.69; *P *< 0.0005). Thirty-two percent (n = 486) were less than 20 years of age; only 4.5% (n = 70) were 60 years or greater. Of all patients, 7% were status Aboriginals, and 11% and 15% received social assistance or an insurance premium subsidy, respectively. Compared with patients hospitalized only once, those with multiple hospitalizations were more likely to be on social assistance (17% vs. 11%; z = -2.11; *P *= 0.04) and Aboriginal (14% vs. 7%; z = -2.83; *P *= 0.005) (Table 1).

**Table 1:** Characteristics of patients hospitalized for acetaminophen overdose *

**Characteristic**	**All patients (n = 1,543)**	**Single hospitalization (n = 1,434)**	**Multiple hospitalizations (n = 109)**	*P*-value **
**Demographics**				
Age, *years*	26 (2–85)	26 (2–85)	27 (14–55)	0.91
Female gender	68% (1,054)	68% (975)	72% (79)	0.20
Aboriginal status	7% (109)	7% (94)	14% (15)	0.005
Social assistance	11% (174)	11% (155)	17% (19)	0.04
Subsidy	15% (226)	15% (215)	10% (11)	0.16
Depression	55% (842)	54% (774)	62% (68)	0.09
Alcohol-related diagnosis	33% (515)	33% (467)	44% (48)	0.01
				
**Circumstance of overdose**				
Intentional	85% (1,427) ^‡^	85% (1,215)	88% (96)	0.73
Accidental	13% (220) ^‡^	13% (193)	11% (12)	
Other	2% (33) ^‡^	2% (26)	1% (1)	

* Data at first hospitalization. All data are medians (interquartile range) or proportions (% [n]).

** For comparison of patients with single versus multiple hospitalizations.

^‡ ^Percentages calculated based on total number of overdoses (n = 1,680).

Depression or at least one alcohol-related diagnosis were recorded in 55% (n = 842) and 33% (n = 515) of the patients, respectively; 17% (n = 270) had both conditions. The most common alcohol-related diagnoses were alcohol abuse (12%), alcohol dependence disorders (11%), and alcohol-related psychiatric disorders (15%). Alcohol-related diagnoses were more common in males (47% vs. 27%; z = -7.52; *P *< 0.0005) and Aboriginals (65% vs. 31%; z = -7.29; *P *< 0.0005), but did not differ according to receipt of social assistance or an insurance premium subsidy. Alcohol-related diagnoses, but not depression, were more common in patients with multiple versus single hospitalizations for acetaminophen overdose (Table 1).

**Figure 1:**
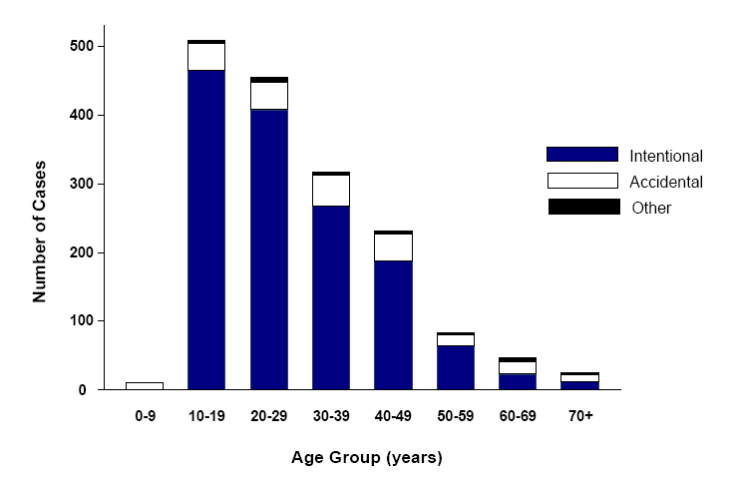
Cases of acetaminophen overdose in the Calgary Health Region by age and intention (1995–2004).

The majority of the overdoses (85%) were intentional. Only 13% were accidental and 2% were homicidal (n = 3) or of undetermined intent (n = 30) (Table 1). The proportion with intentional overdoses decreased with advancing age (chi^2 ^[7 df] = 166.3; *P *= 0.0001; Figure 1). Only 45% (22/49) of overdoses in seniors were intentional versus 86% (1,405/1,631) in younger patients (z = 7.95; *P *< 0.0005). All overdoses in patients under 10 years (n = 11) were accidental.

### Hospitalization rates

**Figure 2:**
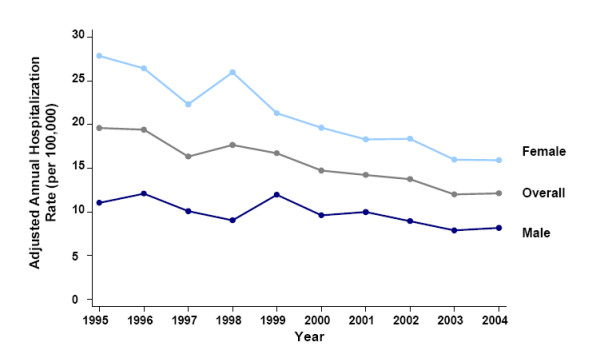
Annual age-adjusted hospitalization rates for acetaminophen overdose by sex (1995–2004).

**Figure 3:**
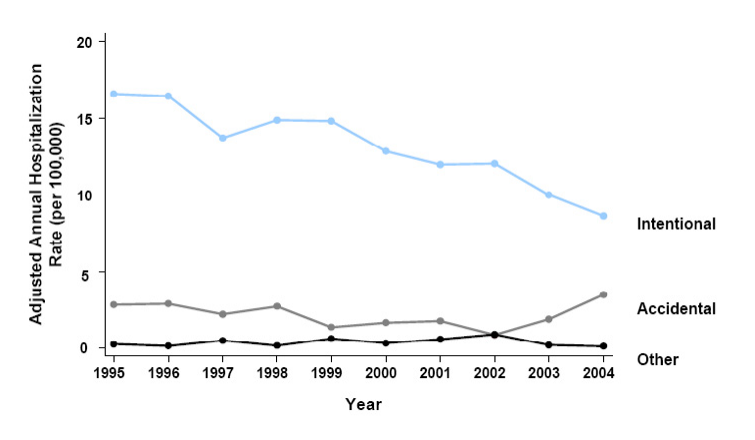
Annual age/sex-adjusted hospitalization rates for acetaminophen overdose by intention and year (1995–2004).

Between 1995 and 2004, the age/sex-adjusted annual hospitalization rate for acetaminophen overdose was 15.5 per 100,000 population. Females were approximately twice as likely to be hospitalized. Age-adjusted rates in females and males were 21.3 and 9.7 per 100,000, respectively (rate ratio [RR]: 2.19; z = 472.16; *P *< 0.0005). Hospitalization rates decreased by 41% between 1995 and 2004 (Figure 2). The adjusted hospitalization rate in 1995 was 19.6 per 100,000 versus 12.1 per 100,000 in 2004. This decline was more marked in females than males (46% vs. 29%). Hospitalization rates declined steadily for intentional overdoses from 16.6 per 100,000 in 1995 to 8.6 per 100,000 in 2004 (2004 vs. 1995: RR 0.49; z = -178.52; *P *< 0.0005) (Figure 3). On the contrary, accidental overdoses decreased during the middle years of the interval and increased back above baseline levels by 2004 (2004 vs. 1995: RR 1.24; z = 26.49; *P *< 0.005) (Figure 3). Overdoses of other causes fluctuated at low levels (Figure 3).

**Figure 4:**
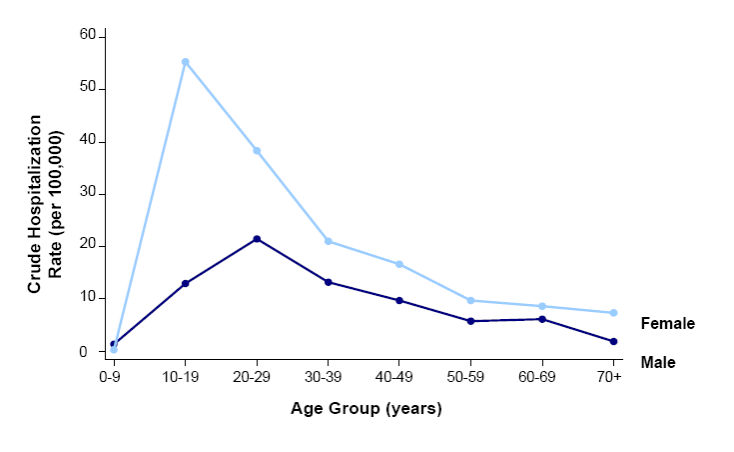
Crude hospitalization rates for acetaminophen overdose by age and sex (1995–2004).

The highest hospitalization rates were observed in the 10–19 and 20–29 year age groups (35.5 and 30.3 per 100,000 population, respectively) (Figure 4). These groups were nearly three-times as likely to have an overdose compared with patients 30 years and older (RR 2.76; z = 648.30; *P *< 0.0005). Excluding patients under 10 years, hospitalization rates were significantly higher in females (Figure 4). The greatest disparity was seen in the 10–19 year age group in whom the crude hospitalization rate was 55.4 per 100,000 in females versus only 13.0 per 100,000 in males (RR 4.24; z = 398.04; *P *< 0.0005).

**Figure 5:**
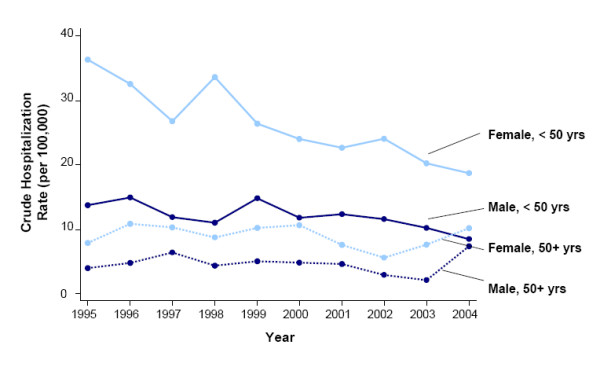
Trends in crude hospitalization rates for acetaminophen overdose by age group (< vs. ≥ 50 years) and sex (1995–2004).

Trends in hospitalization rates for acetaminophen overdose varied by age group (Figure 5). Between 1995 and 2004, rates declined in both males and females under 50 years (2004 vs. 1995: males, RR 0.62 [z = -69.71; *P *< 0.0005]; females, RR 0.51 [z = -148.78; *P *< 0.0005]; overall, RR 0.54; z = -164.13;*P *< 0.0005). In patients 50 years and over, rates tended to fluctuate (Figure 5). Compared with 1995, rates in 2004 were higher in both males and females (2004 vs. 1995: males, RR 1.86 [z = 33.58; *P *< 0.0005]; females, RR 1.29 [z = 17.43; *P *< 0.0005]; overall, RR 1.50 [z = 35.60; *P *< 0.0005).

Aboriginal status and receipt of social assistance were important risk factors for acetaminophen overdose. The RRs of Aboriginal status and social assistance were 4.04 (*P *< 0.0005) and 5.16 (*P *< 0.0005), respectively. Receipt of an insurance premium subsidy was not a significant risk factor (RR 0.97; *P *= 0.74).

## Discussion

In this population-based study, we examined trends in hospitalization rates for acetaminophen overdose in a large Canadian health region. Between 1995 and 2004, the adjusted annual hospitalization rate for acetaminophen overdose was 15.5 per 100,000 population. This rate is approximately one-fifth of that reported in the UK despite legislation limiting sales of acetaminophen in that country[12,27,28]. Although the explanations for this discrepancy are beyond the scope of this study, differences in acetaminophen availability seem unlikely considering the relatively liberal sale of acetaminophen in Canada. Moreover, suicide rates are similar in the UK and Canada[29,30]. Presumably, this discrepancy reflects different methods of suicide in the two countries (eg. more frequent use of firearms, hanging, and suffocation in Canada versus poisoning in the UK)[29,30]. We also report a 41% decline in the annual rate of hospitalization between 1995 and 2004. This decline was more pronounced in females, younger patients (under 50 years), and for intentional overdoses. Our study is at odds with a recent Canadian epidemiologic investigation of acetaminophen overdose[6]. In this analysis of Canada-wide hospital discharge data, Prior *et al*. reported only a slight decrease (~10%) in hospitalization rates for acetaminophen overdose between 1995 and 2001 (vs. 30% in our study). The purpose of this study was to examine the impact of the lifting of place-of-sale restrictions in 1999 on rates of acetaminophen overdose. Provincial data for Alberta was aggregated with that of other provinces (Nova Scotia and Prince Edward Island) that did not have restrictions prior to this time point. Therefore, data specific to our health region or Alberta cannot be extracted from this report. Of pertinence is that overdose rates in our health region appear to differ from other regions, even within Alberta. For example, Colman *et al*. reported much lower rates of emergency department visits for self-inflicted injuries in Calgary compared with Edmonton (the other major city in the province) during the same time period[31]. This finding likely reflects differences in the underlying populations. Since low SES appears to be associated with acetaminophen overdose (see below), the relative prosperity of the CHR due to its high concentration of petroleum companies may account for the lower rates that we observed.

The majority of the data from other countries supports our observation. Although US data is limited, Nourjah *et al*. reported a 10% decline in acetaminophen-related 'poison control calls' to centers involved in the Toxic Exposure Surveillance System between 1997 and 2001[13]. Similarly, Turvill *et al*. reported declines of 21% and 64% in all acetaminophen overdoses and severe overdoses, respectively, presenting to the Royal Free Hospital in London between 1995 and 2002[28]. In Scotland, Bateman *et al*. reported an increase in hospitalizations from 1990 to 1997 followed by an approximate 20% decrease between 1997 and 1999 (2000–2004 data was not reported)[12]. Hughes *et al*. also reported a fall in hospital admissions for acetaminophen overdose in Birmingham between 1995 and 1999[32]. These reductions have been attributed to legislation limiting the sale of acetaminophen in the UK The fall in hospitalization rates in our region is somewhat surprising since efforts to reduce acetaminophen overdose, including package size restrictions, have not been undertaken. Colman *et al*. actually reported an *increase *in visits to emergency departments in Alberta for self-inflicted injuries during this time period (but trends in acetaminophen overdose were not reported)[31]. In addition, Canadian suicide rates have remained stable during the past two decades[29]. Presumably this conflicting data relates to shifting trends in methods of suicide[29]. Huchcroft *et al*. observed a decline in self-poisoning deaths in Canadian females, but an increase in suicides due to hanging, strangulation and suffocation in males[33]. An alternative explanation is that thresholds for hospitalizing patients with acetaminophen overdose have become more stringent. Increasing demand for hospital beds and greater experience managing these patients are possible explanations. Because our data does not represent all incident cases, we cannot confirm these speculations.

Hospitalization rates for accidental acetaminophen overdose appeared to rise during the latter years of our study following an initial decline between 1995 and 2002. Such 'therapeutic misadventures' [34,35] occurred in 13% of our study population. This finding is in keeping with data from the US ALF Study Group reporting that a striking 50% of ALF cases due to acetaminophen were accidental[2]. Based on this data, it has been estimated that approximately 500 ALF cases and 150 deaths attributable to unintentional overdoses occur annually in the US[36]. Since accidental ingestions have been linked with a greater risk of hepatotoxicity, [3,37-39] our observation of a recent increase is of public health importance. Currently over 100 products containing sometimes large amounts of acetaminophen are available over-the-counter, and many patients (and physicians) are unaware of their acetaminophen content[36]. The observed increase in hospitalization rate coincides with the availability of extended release acetaminophen preparations in Canada (since 1999); misinformation about the proper dosing of these formulations may have played a role. Our data emphasizes the necessity of educational initiatives regarding the safe use of acetaminophen and clear labeling of medications with their acetaminophen content so that this trend does not continue.

A major strength of our data is the examination of population-based, sociodemographic risk factors for acetaminophen overdose. As reported in other studies, females, especially those in their teenage years and twenties, are at greatest risk[6,12,28,32,40-44]. Hospitalization rates are four to five-fold higher among individuals who require social assistance and status Aboriginals. High rates of suicidal behaviour have been reported in the Aboriginal communities of Canada and other countries,[45,46]. emphasizing the necessity of suicide prevention strategies in this population. Presumably sociodemographic factors contribute to this risk, but this could not be examined specifically due to coding methods among Aboriginals in our databases. The high rate of alcohol-related diagnoses in our study cohort (33%), particularly among status Aboriginals (64%), likely contributed to the risk of acetaminophen overdose in these subgroups[47].

Another strength of our study is the use of population-based data from a large geographic region. Many prior studies have originated in referral centres, including liver transplant units, and are prone to selection bias[2-5,38]. For example, data from the US ALF Study Group suggests that the proportion of ALF cases due to acetaminophen overdose is on the rise[2]. However, since denominator data is not available in this type of study due to the recruitment methods, population-based studies such as our own are necessary to truly appreciate trends in rates of acetaminophen overdose. Moreover, part of the explanation for the apparently increasing rates in this study (versus the *decrease *that we observed) is that the proportion of ALF cases due to viral hepatitis has fallen presumably as a result of widespread vaccination. This shift in etiologies has likely led to an overemphasis of the importance of acetaminophen overdose in current data.

Our study has several limitations. First, the validity of coding suicidal intent in patients with acetaminophen overdose has not been validated. However, studies of other conditions have suggested that E-codes provide a reliable indication of suicidal intent[48,49]. For example, LeMier *et al*. reported 95% agreement between E-codes obtained from administrative data and medical record review for defining suicidal intent in a variety of injuries including poisonings, falls, and firearm incidents. For poisonings specifically, agreement was 87%[48]. In another study of adult subscribers to a health maintenance organization in California, medical record reviews confirmed that 86% of hospitalizations assigned "intentional" E-codes were suicide attempts[49]. An additional limitation of our study is the reliance on discharge data to identify only hospitalized cases, which clearly underestimates the true incidence of acetaminophen overdose. Patients who didn't seek medical attention or weren't hospitalized would not have been captured by our search strategy. However, we have identified the most clinically relevant cases at the highest risk of adverse outcomes and consumption of health care resources.

## Conclusion

Rates of acetaminophen overdose, particularly intentional ingestions, have fallen in our Canadian health region between 1995 and 2004. Young patients, especially females, Aboriginals, and recipients of social assistance, are at highest risk.

## Abbreviations

AHCIP, Alberta Health Care Insurance Plan; ALF, acute liver failure; CHR, Calgary Health Region; RR, rate ratio; SES, socioeconomic status

## Competing interests

The author(s) declare that they have no competing interests.

## Authors' contributions

Dr. Myers conceived the study idea, performed all statistical analyses, and drafted the manuscript. B. Li and A. Fong performed data extraction and revised the manuscript critically for important intellectual content. Drs. A. Shaheen and H. Quan assisted with statistical analysis and revised the manuscript critically for important intellectual content. All authors read and approved the final version of the manuscript. Dr. Myers is the guarantor of the study.

## Pre-publication history

The pre-publication history for this paper can be accessed here:


